# Diverging trends in alcohol use and mental health in Australian adolescents: A cross‐cohort comparison of trends in co‐occurrence

**DOI:** 10.1002/jcv2.12241

**Published:** 2024-05-23

**Authors:** Tim Slade, Cath Chapman, Jillian Halladay, Matthew Sunderland, Anna Smout, Katrina E. Champion, Nicola C. Newton, Maree Teesson

**Affiliations:** ^1^ The Matilda Centre for Research in Mental Health and Substance Use The University of Sydney Sydney New South Wales Australia; ^2^ Turner Institute for Brain and Mental Health Monash University Melbourne Victoria Australia

**Keywords:** adolescent, alcohol, anxiety, depression, psychological distress, trends

## Abstract

**Background:**

In recent years, psychological distress in Western countries has rapidly increased among older adolescents while alcohol use has declined, though little is known about younger adolescents. It is also unclear if and how these trends relate to co‐occurring alcohol use and distress. This study sought to examine temporal changes in the prevalence of distress, alcohol use, and their co‐occurrence among young Australians.

**Methods:**

This study used data from 13,388 youth in their early teens (aged 12–14). Differences in the prevalence of high psychological distress (Kessler‐6 ≥ 13), any alcohol use (standard drink in past 3/6 months), and their co‐occurrence across cohorts (2007, 2012, 2014, 2019) were tested through log‐binomial regression models. Changes in co‐occurrence across cohorts were tested with cohort‐by‐alcohol interactions predicting distress, and vice‐versa. Differential trends by sex were evaluated.

**Results:**

From 2007 to 2019, the prevalence of high distress more than doubled (4.6%–13.5%) while alcohol use decreased by ∼90% (11.8%–3.1%). Distress increased more‐so among females, while alcohol use decreased more‐so among males. The prevalence of high distress was significantly greater among adolescents who used alcohol compared to those who had not (>2 times higher), with this co‐occurrence remaining consistent across cohorts.

**Conclusions:**

Psychological distress appears to be increasing similarly among youth in their early teens who do and do not use alcohol. At the same time, alcohol use is decreasing similarly among youth with and without distress. While alcohol use does not appear to be a driver of increases in distress, rates of co‐occurring alcohol use and distress remain high. Addressing co‐occurrence and distress‐specific mechanisms remain necessary.

**Key points**

**What's known?**

- Generational changes in depression, anxiety and alcohol use are emerging with yet little is known about trends in the association between mental health problems and alcohol use.

**What's new?**

- Psychological distress among young Australian adolescents has doubled in recent years, particularly among females, and alcohol use decreased by 90%, with a larger decrease among males than females.
- The association between psychological distress and alcohol use has remained stable over time, both in females and males.

**What's relevant?**

- While alcohol does not appear to be driving the recent increases in psychological distress, identifying ways to address reasons for increasing distress as well as reasons for co‐occurring alcohol and distress are critical.

## INTRODUCTION

Substance use and emotional distress are substantial societal health challenges. Internationally, rates of adolescent anxiety (Blomqvist et al., 2019), depression (Blomqvist et al., 2019; Daly, 2022; Keyes et al., 2019), and suicidality (Twenge et al., 2019) have been increasing over the past 15–20 years, while at the same time common substance use has largely been declining or plateauing (Grucza et al., 2018; Reitsma et al., 2021; Vashishtha et al., 2021). Notably, alcohol use—the most commonly used substance—has declined across high income countries (Vashishtha et al., 2021). In Australia, while adolescent data are limited, rates of anxiety, depression, and distress are on the rise, particularly among young adult females (Butterworth et al., 2020; Enticott et al., 2022). In contrast, the rates of alcohol use have been declining, more‐so among males and largely due to higher rates of abstention (Livingston et al., 2022). Most of the existing studies are based on samples of older adolescents, largely examining these trends independent of one another. As such, it is unclear whether these trends are also observed among younger adolescents (i.e. <14 years of age), where alcohol use is not a normative behaviour, and if and how these changes relate to each other (Halladay et al., 2023).

While these trends are typically examined separately, similar influencing factors are often discussed. Some attribute changes to the rise of social media (De Looze et al., 2018). Others point to concurrent changes in health‐related behaviours such as declines in sleep quality, increases in physical inactivity and sedentary behaviour (Patalay & Gage, 2019), and declines in delinquent behaviours generally (Grucza et al., 2018). Changes may also be related to shifts in social acceptability, perceived harm, or stigma (Caluzzi et al., 2022), or to the differential effects of prevention strategies to address these concerns (Tremblay et al., 2020). Importantly, we do not yet understand why these changes are happening concurrently in different directions, particularly as emotional distress and substance use commonly co‐occur (Teesson et al., 2009) and share common risk factors (Slade & Watson, 2006; Sunderland et al., 2015). It should be noted, however, that there is evidence to suggest the relationship between heavy alcohol use and emotional distress may not be as strong in younger adolescents compared to those at other life stages (Pape & Norstrom, 2016).

To examine trends in co‐occurrence, we require large, comparable samples of adolescents assessed across significant stretches of historical time that use similar measures for alcohol use and emotional distress. While current explorations are limited, there are several possibilities for how changing trends are related to co‐occurring problems (Halladay et al., 2023). One possibility is that the relationship has ‘hardened’ or strengthened over time such that as alcohol use becomes less common, it carries a ‘higher risk’ of emotional distress among the smaller pool of adolescents who are drinking. It is also possible that the strong relationship between adolescent alcohol use and emotional distress has remained the same or ‘consistent’ over time. Alternatively, there may be a weakening or ‘decoupling’ of the relationship. Each of these possible changes in joint‐trends suggests shifts (or consistencies) in the balance of shared risk and protective factors over time, partly reflecting and partly forecasting changes in the underlying pathways to, and risk factors for, mental health and substance use problems (Halladay et al., 2023). Being aware of these changes has important implications for our understanding of developmental psychopathology as well as prevention and early intervention responses.

The declines in alcohol have been largely attributed to later age of initiation, with the average age of onset among young Australians increasing from 14.5 in 2001 to 16.2 in 2019 (Australian Institute of Health and Welfare, 2020). Earlier age of alcohol use initiation, particularly by 14, is associated with more substance‐ and mental health‐related problems including greater rates of suicidality (Baiden et al., 2019). Few studies have examined joint‐trends in associations between alcohol use and mental health among youth in their early teens (Halladay et al., 2023). Gage and Patalay (2021) compared associations between alcohol use and depressive symptoms from two UK birth cohorts at age 14 in 2005–2006 and 2015–2017. They found lifetime alcohol use to be more strongly related to depressive symptoms in 2015–2017 compared to 2005–2006, though there was no change in the magnitude of association between depressive symptoms and heavy episodic drinking (5+ drinks) (Gage & Patalay, 2021). Other studies exploring joint‐trends in older and/or broader samples of adolescents have found equivocal results, reporting either strengthened (Ng Fat et al., 2018; Pape & Rossow, 2021; Torikka et al., 2017), consistent (Kahn & Wilcox, 2022), or weakened (Askari et al., 2021; Keyes et al., 2020) associations between alcohol and mental health concerns (mainly depression).

Within this context, several unanswered questions remain. During early adolescence, is the relationship between alcohol and mental health strengthening, staying consistent, or weakening for recent cohorts? Does this trend match what is seen in broader, and older cohorts of adolescents? Is this trend consistent across samples and countries? And are these changes occurring to the same degree for males and females? Several countries, for example, have reported evidence that increases in rates of depression, anxiety, self‐harm and psychological distress among recent cohorts of adolescents are driven by greater increases among females than males (Blomqvist et al., 2019; Keyes et al., 2019), whilst others have indicated that cohort changes in alcohol and other drug use and related harms are being driven by decreases among adolescent males rather than females (Pape et al., 2018; Slade et al., 2016).

The present study addresses current gaps in the literature by examining the relationship between alcohol use and psychological distress in a unique, large contemporary dataset of Australian youth in their early teens (∼12–14 years of age), representing birth cohorts from 1994 to 2007. Specifically, it sought to (1) examine changes in the prevalence of any recent alcohol use and psychological distress over time, (2) determine whether the strength of the association between alcohol use and psychological distress has changed over time, and (3) determine whether any observed changes in prevalence or association are different for males and females. Given the equivocal findings of previous research we have not specified any hypotheses.

## METHODS

### Sample

Data came from adolescents aged 12 to 14 participating in four school‐based prevention trials; the Climate Schools study (CS) (Newton et al., 2009), the Climate and Preventure study (CAP) (Newton et al., 2012), the Climate Schools Combined study (CSC) (Teesson et al., 2014), and the Health4Life study (H4L) (Teesson et al., 2020). Baseline data from each trial (i.e., before interventions were delivered) were used to create four cohorts of data collected in 2007, 2012, 2014 and 2019. These four cohorts collectively span a 13‐year socio‐historical period, reflecting experiences of adolescents born between 1994/95, 1999/2000, 2001/02, 2006/07. When combined, the present sample (*N* = 13,388; 49.6% female) contains adolescents from 178 schools across four different states of Australia. Seven schools participated in more than one study and as such, feature in two cohorts, however no single adolescent contributed more than one observation. Information pertaining to the Index of Community Socio‐Educational Advantage (ICSEA) for the cohorts and total sample are provided in Table 1 to further contextualise the sample. ICSEA scores provide information about the socio‐educational circumstances of students attending a given school and are calculated using measures of parent occupation, parent education, geographical location, and proportion of Indigenous students who attend the school (MySchool, 2016). Possible ICSEA scores range from 500 (extremely educationally disadvantaged) to 1300 (very educationally advantaged) with scores of 1000 classified as average (SD = 100).

**TABLE 1:** Sample characteristics for four studies included in analysis.

Name	Cohort						ICSEA^a^		
	Birth cohort	Year of measurement	*N* (schools)	*N* (participants)	Sex (% female)	School type	Range	Percentile	Median
Climate schools (CS)	1994/1995	2007	10	757	40.3%	Independent (10)	NA	NA	NA
Climate and PreVenture (CAP)	1999/2000	2012	26	2180	42.6%	Catholic (5)	982–1200	40–99	1132
						Government (9)			
						Independent (12)			
Climate schools combined (CSC)	2001/2002	2014	71	6253	55.1%	Catholic (11)	909–1243	10–99	1044
						Government (41)			
						Independent (19)			
Health4Life (H4L)	2006/2007	2019	71	4198	47.0%	Catholic (12)	900–1202	9–99	1068
						Government (24)			
						Independent (35)			
Total sample	1994–2007	2007–2019	178^b^	13,388	49.6%	Catholic (28)	900–1243^c^	9–99^c^	1063.5^c^
						Government (71)			
						Independent (76)			

^a^ Possible ICSEA scores range from 500 (extremely educationally disadvantaged) to 1300 (very educationally advantaged) with scores of 1000 classified as average (SD = 100).

^b^ Seven schools participated in two cohorts.

^c^ Estimated based on cohorts with available data.

### Measures

The Kessler 6 (K6) was used across all studies to assess psychological distress. This self‐report scale measures the frequency of six mood and anxiety symptoms (1. Feeling nervous, 2. Feeling hopeless. 3. Feeling restless or fidgety, 4. Feeling so depressed nothing could cheer you up, 5. Feeling that everything was an effort, 6. Feel worthless) in the past 30 days on a five‐point scale (0 = none of the time, 1 = a little of the time, 2 = some of the time, 3 = most of the time, 4 = all of the time). Total K6 scores range from 0 to 24 with high scores indicating higher psychological distress. The K6 has demonstrated good performance when predicting scores on related measures of psychopathology (Mewton et al., 2016). For the main analysis, the commonly used diagnostic cut‐point of 13 or more determined a positive screen for high psychological distress (Kessler et al., 2002). A yes/no question asking whether the students had consumed at least one full standard drink (10 mg alcohol) in the past 6 months (past 3 months in 2007) was used to define any alcohol use. Those that answered yes were asked about frequency of heavy episodic drinking, defined as consuming five or more drinks on one occasion, coded as yes/no for sensitivity analyses.

### Statistical analysis

Prior to main analyses, the differential item functioning (DIF) of K6 items across the cohorts was explored to confirm appropriateness of cross‐cohort comparisons. Applying a method combining logistic ordinal regression and item response theory (Choi et al., 2011), items demonstrated only trivial levels of DIF across cohorts. This supports cross‐cohort comparison of K6 scores. See Supporting Information S1 for details.

The prevalence of high psychological distress, any drinking, and the co‐occurrence of the two were computed by cohort and sex. Differences in prevalence of each outcome across cohorts were tested by fitting log binomial regression models to the combined dataset. Log binomial regression was preferred over logistic regression to directly model the probability (or prevalence) rather than the odds of the outcome conditional on the model predictors. Prevalence ratios (PR) and their 95% confidence intervals are presented. A model containing a main effect for cohort (treated categorically with the 2007 cohort as the reference) tested whether the prevalence of each outcome changed across cohorts (i.e., over historical time). A model containing a main effect for sex (male as the reference) tested whether the prevalence in the total dataset was different for males and females. A model containing main effects for cohort and sex and a cohort‐by‐sex interaction determined whether any changes in prevalence over time were different for males and females. PRs of 1.32 are considered small, 1.91 medium, and 2.48 large population effects (Matthay et al., 2021).

To examine the changing *association* between psychological distress and alcohol use across cohorts, two further log binomial regression models were estimated, in the total sample as well as in the male and female subgroups. In the first model, high psychological distress was the outcome variable and the predictors were cohort, any alcohol use, and a cohort‐by‐alcohol use interaction. In the second model, any alcohol use was the outcome variable and the predictors were cohort, high psychological distress, and a cohort‐by‐high distress interaction. Sensitivity analyses were carried out in the full sample using heavy episodic drinking in place of any alcohol use.

Item level missing data meant that when alcohol use was considered alone the analysed sample consisted of 13,290 respondents. When psychological distress was considered alone the analysed sample consisted of 12,823 respondents. When examining the relationship between the two the analysed sample consisted of 12,774 respondents. Alcohol use did not differ among those with versus without valid psychological distress data (*X* ^2^ = 0.24, *p* = 0.622). Similarly, high psychological distress did not differ among those with versus without valid alcohol use data (*X* ^2^ = 0.09, *p* = 0.764). Analyses were carried out in Stata v16.1 using the *glm* procedure specifying a binomial outcome distribution with a log link function. All standard errors, significance tests, and confidence intervals were adjusted for school‐level clustering.

## RESULTS

The prevalence of high psychological distress and alcohol use across cohorts and sex are provided in Table 2 (graphs in Supporting Information S1). In the combined sample, the prevalence of high psychological distress was 10.3% (7.5% in males, 13.1% in females) and the prevalence of any alcohol use was 6.7% (8.2% in males, 5.3% in females). The results of the log binomial regression analyses demonstrated a significant cohort effect for both high psychological distress (chi‐sq = 75.93, df = 3, *p* < 0.001) and any alcohol use (chi‐sq = 127.84, df = 3, *p* < 0.001) with PRs indicating an increase in high psychological distress and a decrease in any alcohol use over time. There was a significant sex‐by‐cohort interaction for both high psychological distress (chi‐sq = 11.60, df = 3, *p* = 0.0089) and any alcohol use (chi‐sq = 9.46, df = 3, *p* = 0.0238) demonstrating that the increase in distress over cohorts was more apparent in females while the decrease in alcohol use over cohorts was more pronounced in males.

**TABLE 2:** Prevalence of high psychological distress and any alcohol use by sex and cohort and prevalence ratios across cohorts.

	Prevalence of high psychological distress (*N* = 12,823)	Prevalence ratio across cohorts^a^	Prevalence of any alcohol use (*N* = 13,290)	Prevalence ratio across cohorts^a^
	% (95% CI)	PR (95% CI)	% (95% CI)	PR (95% CI)
Total sample				
2007 cohort (ref)	4.6 (3.6, 5.7)	‐	22.8 (16.7, 28.9)	‐
2012 cohort	8.7 (7.5, 9.8)	1.9 (1.4, 2.4)	10.3 (8.7, 11.8)	0.5 (0.3, 0.6)
2014 cohort	9.4 (8.2, 10.7)	2.0 (1.6, 2.6)	6.0 (4.4, 7.6)	0.3 (0.2, 0.4)
2019 cohort	13.5 (12.0, 15.1)	2.9 (2.3, 3.7)	3.1 (2.4, 3.9)	0.1 (0.1, 0.2)
Total sample	10.3 (9.5, 11.2)		6.7 (5.7, 7.9)	
Males				
2007 cohort (ref)	4.6 (3.0, 6.3)	‐	27.9 (21.7, 34.0)	‐
2012 cohort	7.7 (6.7, 8.7)	1.7 (1.1, 2.4)	10.3 (8.4, 12.1)	0.4 (0.3, 0.5)
2014 cohort	6.0 (4.9, 7.1)	1.3 (0.9, 1.9)	7.3 (5.1, 9.4)	0.3 (0.2, 0.4)
2019 cohort	10.0 (8.2, 11.9)	2.2 (1.5, 3.2)	4.2 (3.1, 5.4)	0.2 (0.1, 0.2)
All males	7.5 (6.7, 8.4)		8.2 (6.5, 9.8)	
Females				
2007 cohort (ref)	4.6 (3.3, 5.9)	‐	15.4 (9.7, 21.2)	‐
2012 cohort	10.0 (7.8, 12.1)	2.2 (1.5, 3.1)	10.3 (7.3, 13.3)	0.7 (0.4, 1.1)
2014 cohort	12.2 (10.4, 14.1)	2.6 (1.9, 3.6)	5.0 (3.5, 6.4)	0.3 (0.2, 0.5)
2019 cohort	17.3 (15.5, 19.1)	3.7 (2.8, 5.0)	1.9 (1.3, 2.6)	0.1 (0.1, 0.2)
All females	13.1 (11.8, 14.4)		5.3 (4.1, 6.4)	

*Note*: High psychological distress defined as a score of 13+ on the K6. Any alcohol use defined as at least one standard drink of alcohol in the past 6 months (past 3 months for the 2007 cohort).

^a^ Ratio of prevalence in the 2012, 2014 and 2019 cohorts compared to the 2007 cohort.

Across all cohorts, for both males and females, the prevalence of high psychological distress was significantly greater among those who had used alcohol compared to those who had not (see Table 3). While the point estimates of PRs for the relationship between alcohol use and psychological distress suggested some weakening across cohorts (i.e., the ratio of the prevalence of high psychological distress among those with vs. without alcohol use was numerically lower in all cohorts when compared to 2007), and the PR for 2019 versus 2007 among females was significant (*p* = 0.025), modelling of the interaction between cohort and any alcohol use provided little evidence that PRs differed meaningfully across cohorts, both in the total sample (chi‐sq = 5.35, df = 3, *p* = 0.1482) and separately in males (chi‐sq = 0.87, df = 3, *p* = 0.8339) and females (chi‐sq = 5.28, df = 3, *p* = 0.1527). Further, point estimates of PRs across all years and groups were moderate to large in magnitude (PRs 2.3–6.4), suggesting alcohol use was consistently and strongly associated with elevated levels of psychological distress.

**TABLE 3:** Prevalence of high psychological distress by sex, cohort and presence versus absence of alcohol use (*N* = 12,774).

	Prevalence of high psychological distress in those *with* alcohol use	Prevalence of high psychological distress in those *without* alcohol use	Prevalence ratio within cohorts^a^	Prevalence ratio across cohorts^b^
	% (95% CI)	% (95% CI)	PR (95% CI)	PR (95% CI)
Total sample				
2007 cohort (ref)	11.4 (6.5, 16.3)	2.3 (1.1, 3.4)	4.9 (2.0, 11.9)	‐
2012 cohort	21.8 (17.1, 26.5)	7.2 (6.1, 8.4)	3.0 (2.3, 4.0)	0.6 (0.3, 1.5)
2014 cohort	26.9 (21.2, 32.6)	8.4 (7.2, 9.5)	3.2 (2.6, 4.0)	0.7 (0.3, 1.6)
2019 cohort	30.2 (21.8, 38.7)	13.0 (11.5, 14.4)	2.3 (1.8, 3.0)	0.5 (0.2, 1.1)
Total sample	23.1 (19.8, 26.5)	9.4 (8.5, 10.2)		
Males				
2007 cohort (ref)	9.9 (3.8, 16.0)	2.2 (0.2, 4.3)	4.4 (1.0, 19.9)	‐
2012 cohort	17.5 (12.4, 22.6)	6.6 (5.7, 7.4)	2.7 (1.9, 3.7)	0.6 (0.1, 2.5)
2014 cohort	15.1 (8.7, 21.5)	5.3 (4.2, 6.3)	2.9 (1.8, 4.5)	0.6 (0.2, 2.8)
2019 cohort	23.1 (13.9, 32.2)	9.4 (7.8, 11.1)	2.4 (1.7, 3.4)	0.6 (0.1, 2.3)
All males	15.8 (12.2, 19.4)	6.8 (5.9, 7.6)		
Females				
2007 cohort (ref)	15.2 (10.6, 20.0)	2.4 (1.5, 3.3)	6.4 (3.2, 12.5)	‐
2012 cohort	27.5 (21.2, 33.7)	8.0 (5.8, 10.3)	3.4 (2.7, 5.1)	0.5 (0.3, 1.1)
2014 cohort	41.0 (32.2, 49.7)	10.8 (9.0, 12.6)	3.8 (3.0, 4.9)	0.6 (0.3, 1.1)
2019 cohort	47.4 (31.3, 63.3)	16.7 (14.9, 18.5)	2.8 (2.0, 4.1)	0.4 (0.2, 0.9)
All females	34.5 (28.9, 40.2)	11.8 (10.8, 13.2)		

*Note*: High psychological distress defined as a score of 13+ on the K6. Any alcohol use defined as at least one standard drink of alcohol in the past 6 months (past 3 months for the 2007 cohort).

^a^ Ratio of prevalence in those with alcohol use compared to those without alcohol use in each cohort.

^b^ Ratio of prevalence in the 2012, 2014 and 2019 cohorts compared to the 2007 cohort.

Results were similar when considering any alcohol use as the outcome. Within each cohort the prevalence of any alcohol use was consistently and significantly higher among those who were high versus not high in psychological distress (see Table 4). There was little evidence that the association between any alcohol use and high psychological distress was different across the cohorts in the total sample (chi‐sq = 1.71, df = 3, *p* = 0.6339) as well as separately in males (chi‐sq = 0.21, df = 3, *p* = 0.9760) and females (chi‐sq = 2.23, df = 3, *p* = 0.5257). Again, PRs were consistently moderate to large (PRs 2.3–5.0) suggesting psychological distress has remained strongly associated with early alcohol use.

**TABLE 4:** Prevalence of any alcohol use by sex, cohort and high versus low psychological distress (*N* = 12,774).

	Prevalence of any alcohol use in those *with* high psychological distress	Prevalence of any alcohol use in those *without* high psychological distress	Prevalence ratio within cohorts^a^	Prevalence ratio across cohorts^b^
	% (95% CI)	% (95% CI)	PR (95% CI)	PR (95% CI)
Total sample				
2007 cohort (ref)	59.4 (39.0, 79.8)	21.2 (15.1, 27.3)	2.8 (1.8, 4.4)	‐
2012 cohort	25.3 (18.9, 31.7)	8.6 (7.3, 10.0)	2.9 (2.2, 3.8)	1.0 (0.6, 1.7)
2014 cohort	16.8 (12.9, 20.7)	4.8 (3.3, 6.2)	3.5 (2.7, 4.6)	1.3 (0.8, 2.1)
2019 cohort	7.2 (5.0, 9.5)	2.6 (1.9, 3.4)	2.8 (2.0, 3.9)	1.0 (0.6, 1.7)
Total sample	15.1 (12.6, 17.7)	5.8 (4.7, 6.9)		
Males				
2007 cohort (ref)	63.2 (32.2, 94.2)	26.3 (20.0, 32.7)	2.3 (1.2, 4.6)	‐
2012 cohort	23.1 (16.2, 29.9)	9.0 (7.4, 10.7)	2.6 (1.8, 3.5)	1.1 (0.5, 2.1)
2014 cohort	18.2 (10.5, 26.0)	6.5 (4.4, 8.7)	2.8 (1.8, 4.4)	1.2 (0.6, 2.5)
2019 cohort	10.0 (6.1, 14.0)	3.7 (2.7, 4.8)	2.7 (1.8, 4.1)	1.1 (0.5, 2.3)
All males	17.4 (13.1, 21.6)	7.5 (5.9, 9.1)		
Females				
2007 cohort (ref)	53.8 (33.0, 74.7)	13.7 (8.8, 18.6)	3.9 (2.7, 5.6)	‐
2012 cohort	27.5 (16.8, 38.2)	8.0 (5.6, 10.5)	3.4 (2.3, 5.0)	0.9 (0.5, 1.4)
2014 cohort	16.3 (11.4, 21.1)	3.3 (2.1, 4.4)	5.0 (3.5, 7.0)	1.3 (0.8, 2.0)
2019 cohort	5.5 (3.0, 7.9)	1.3 (0.1, 1.8)	4.3 (2.3, 8.2)	1.1 (0.5, 2.2)
All females	13.8 (10.6, 17.1)	3.9 (2.9, 5.0)		

*Note*: Any alcohol use defined as at least one standard drink of alcohol in the past 6 months (past 3 months for the 2007 cohort).

^a^ Ratio of prevalence in those with high psychological distress compared to those with low psychological distress in each cohort.

^b^ Ratio of prevalence in the 2012, 2014 and 2019 cohorts compared to the 2007 cohort.

### Sensitivity analyses

When replicating the models with heavy episodic drinking predicting high psychological distress, the association between heavy episodic drinking and high psychological distress across cohorts did not significantly differ in the full cohort (chi‐sq 2.67, df = 3, *p* = 0.4453) or across cohorts for males (PR_2007_ = 4.3 [1.0, 18.8] to PR_2019_ = 2.7 [1.4, 5.0]; chi‐sq 0.86, df = 3, *p* = 0.8361) but there was evidence of weakening for females (PR_2007_ = 12.4 [7.5, 20.6] to PR_2019_ = 4.6 [3.1, 6.7]; chi‐sq 15.76, *p* = 0.0013). When modelling heavy episodic drinking as the outcome, there were no differential associations with high psychological distress across cohorts. To note, confidence intervals were wide given the low prevalence of heavy episodic drinking. See Supporting Information S1 for details.

## DISCUSSION

This paper sought to examine changes in alcohol use and psychological distress in a unique harmonised dataset from four cohorts of youth in their early teens in Australia. From 2007 until 2019 the prevalence of high psychological distress among ∼13‐year‐olds increased from 4.6% to 13.5%, with the prevalence of distress in the most recent cohort being nearly three times higher than in the earliest cohort. At the same time, the proportion of adolescents from the same cohorts reporting alcohol use decreased from 22.8% to 3.1%, such that the prevalence of alcohol use among the 2019 cohort was ∼90% lower compared to the 2007 cohort. When examining alcohol use and high psychological distress together, the association largely remained strong and consistent across cohorts. In the 2019 cohort, nearly one in three adolescents using alcohol reported high psychological distress, while just over one in 10 of those not using alcohol reported high distress. Further, despite some evidence of a moderating effect of sex on the relationship between alcohol use and high psychological distress, high levels of co‐occurring concerns remained common for both males and females across cohorts.

High psychological distress increased across these cohorts of young Australian adolescents, with larger increases among females. To our knowledge, this is the first study documenting rising distress among youth in their early teens in Australia. However, these trends do align with other Australian population studies that have found spikes in emotional concerns among young adults that are more pronounced for females (Butterworth et al., 2020; Enticott et al., 2022). Specific to youth in their early teens, a UK study examining two birth cohorts from 1991‐2 and 2000‐2 found emotional concerns in the more recent cohort to be starting earlier, increasing more steeply, and remaining elevated longer compared to the earlier birth cohort (Armitage et al., 2023). These increases were also more pronounced among females (Armitage et al., 2023). By 2019 in the current study, reflective of adolescents born ∼2006, roughly 1 in 10 males and 1 in 5 females reported high psychological distress. These sex differences highlight the need to consider female‐specific mental health strategies, implemented across multiple settings/sectors such as schools, families and peer groups.

There were large declines in young adolescent alcohol use across cohorts that were more pronounced for young males. This is consistent with other high‐income (Vashishtha et al., 2021) and Australia‐specific (Livingston et al., 2022) epidemiological trends showing substantial declines that are larger for males and driven by abstention or delayed initiation. By 2019 in the current study, overall rates of any alcohol use were low (<5%). Little is known about what is causing these declines though they appear to be partly country‐ and context‐specific and changes in parental practices and monitoring have partly explained trends (Vashishtha et al., 2020). Efforts are needed to maintain this reduction in use and/or delayed initiation, such as scaling up effective, evidence‐based school‐based universal and/or targeted prevention (Newton et al., 2022; Tremblay et al., 2020) and exploring possible benefits of incorporating parent components (Thornton et al., 2018).

Most findings from the present study suggest that despite the diverging trends in psychological distress and alcohol use, their co‐occurrence has remained relatively stable. A previous US‐based study in older adolescents similarly found consistent associations between suicidality and both any alcohol use and heavy episodic drinking between 2011 and 2017 (Kahn & Wilcox, 2022). Conversely, weakened associations between heavy episodic drinking and emotional concerns have been noted in two other US‐based studies with older adolescents that cover a broader historical period from 1991 to 2018 (Askari et al., 2021; Keyes et al., 2020). These studies found complete decoupling (among males and females) where there were no longer any positive associations between heavy episodic drinking and emotional concerns in later years. It is also important to consider that these findings differ from other studies in the UK and Nordics that found strengthened co‐occurrence over time periods beginning prior to 2007 (Gage & Patalay, 2021; Ng Fat et al., 2018; Pape & Rossow, 2021; Torikka et al., 2017), including the study of youth that found strengthening between any alcohol use and depression but consistency in associations with heavy episodic drinking (Gage & Patalay, 2021). It is of note that most of the prior studies have been carried out older samples of adolescents; thus, there may be development factors driving these differences (Pape & Norstrom, 2016).

Given associations between alcohol use and psychological distress largely stayed the same across cohorts, this suggests the increases in distress are occurring at similar rates for youth in their early teens who do or do not use alcohol and decreases in alcohol use are similar for those with and without distress. Any contextual risk or protective factors related to the co‐occurrence of alcohol use and psychological distress would have remained relatively balanced across cohorts, with escalations in distress being driven by new or strengthened unique risk factors (Halladay et al., 2023). The key insight gleaned from this study is that alcohol use, and/or related contextual risk factors, do not appear to be directly contributing to escalations in distress among youth in their early teens. Thus, the ongoing pursuit to identify the drivers of rising psychological distress may benefit from focusing on factors that would equally impact adolescents who do and do not engage in alcohol use. These factors may include shifts in academic and work related pressures (‘hustle‐culture’) (Burgess et al., 2022), anxiogenic environments (climate, financial, and political crises) (Frasquilho et al., 2016; Grant et al., 2023), general health behaviours (exercise, sleep, diet) (Kreski et al., 2022), and the impacts of social media and changes in how youth connect with others (De Looze et al., 2018). Further work is needed to identify the contextual risk factors driving the changes in psychological distress across adolescent cohorts particularly during and following the COVID‐19 pandemic.

The main strength of this work is the harmonisation of data from over 13,000 youth across four states spanning 13 years of historical time from four large, contemporary studies that used similar measures. There are however several limitations. Firstly, while each study used similar recruitment strategies, the states and types of schools differed across studies and the samples were not intended to be representative of all adolescents. Thus, there may be unmeasured demographic or contextual variables impacting estimates. The 2007 study had a smaller sample relative to other studies contributing to wider confidence intervals. The 2007 study also used a 3‐month timeframe for the measurement of alcohol use and hence the observed declines in alcohol use are, in fact, likely smaller than would have been observed if a 6‐month timeframe had been used. Due to measurement (un)availability: (1) past 6 months, rather than lifetime, alcohol use was used as the primary variable (noting that given age at onset of alcohol use, differences between past 6 months and lifetime estimates are likely to be small; Australian Institute of Health and Welfare, 2020), and (2) whether moderation effects of sex were related to biological sex and/or sociocultural gender identity could not be explored given gender was not captured consistently at baseline. Lastly, the data presented here are cross‐sectional. It will be important to examine these relationships longitudinally.

## CONCLUSIONS

In this study of over 13,000 young Australian adolescents between 2007 and 2019, rates of high psychological distress doubled while alcohol use declined by ∼90%. Despite diverging independent trends, the prevalence of psychological distress among adolescents using alcohol stayed the same over time. Adolescents using alcohol consistently reported over two times the prevalence of high psychological distress compared to adolescents not using alcohol. While psychological distress rose more‐so among females and alcohol use declined more‐so for males, the prevalence of co‐occurrence remained consistent across time for both female and male adolescents. Overall, psychological distress appears to be increasing similarly among adolescents who do and do not use alcohol. While alcohol use does not appear to be a driver of increases in psychological distress observed in recent years, rates of co‐occurring alcohol use and distress remain high, and thus efforts are still needed to address both co‐occurrence and distress‐specific mechanisms.

## AUTHOR CONTRIBUTIONS

**Tim Slade**: Conceptualization; data curation; formal analysis; funding acquisition; investigation; methodology; project administration; resources; supervision; writing – original draft; writing – review & editing. **Cath Chapman**: Conceptualization; data curation; formal analysis; funding acquisition; investigation; methodology; resources; supervision; writing – original draft; writing – review & editing. **Jillian Halladay**: Conceptualization; investigation; methodology; writing – review & editing. **Matthew Sunderland**: Conceptualization; data curation; formal analysis; funding acquisition; investigation; methodology; writing – original draft; writing – review & editing. **Anna Smout**: Data curation; investigation; project administration; writing – original draft; writing – review & editing. **Katrina E. Champion**: Data curation; investigation; writing – original draft; writing – review & editing. **Nicola C. Newton**: Conceptualization; data curation; investigation; methodology; project administration; resources; writing – original draft; writing – review & editing. **Maree Teesson**: Conceptualization; data curation; funding acquisition; investigation; methodology; project administration; resources; writing – original draft; writing – review & editing.

## CONFLICT OF INTEREST STATEMENT

MT and NN are co‐founders and Directors of Climate Schools Pty Ltd and OurFutures Institute Ltd, a Not‐For‐Profit established to disseminate evidence‐based prevention programs. The other authors have declared that they have no competing or potential conflicts of interest.

## ETHICAL CONSIDERATIONS

Each trial had ethical approval from relevant committees including (where applicable) the University of New South Wales, University of Sydney, Curtin University, University of Queensland, and Queensland University of Technology Human Research Ethics Committees, as well as the NSW, Queensland, and Western Australian Department of Education and Training and several Catholic Diocese and Education committees.

## Supporting information

Supporting Information S1

## Data Availability

The data that support the findings of this study are available on request from the corresponding author. The data are not publicly available due to privacy or ethical restrictions.
