# Supplementary material for: Diverging trends in alcohol use and mental health in Australian adolescents: A cross‐cohort comparison of trends in co‐occurrence
Source: JCPP Adv. 2024 May 23;4(3):e12241. doi: 10.1002/jcv2.12241 (PMC11472813; doi:10.1002/jcv2.12241)
Supplement: Supplementary file 1 — Supporting Information S1 [file JCV2-4-e12241-s001.docx]

**Supplementary Materials Trends**

**S1. K6 Differential Item Functioning**

Before carrying out the main analysis we examined the differential item functioning (DIF) of individual K6 items across the four cohorts. The presence of DIF would suggest that external factors, other than true levels of psychological distress, influence participant responses to the K6 items across the four cohorts, making cross-cohort comparisons difficult. We applied a method combining logistic ordinal regression and item response theory (Choi et al., 2011). Briefly, for each item, the method uses a series of three nested proportional-odds logistic regression models with each K6 item acting as the dependent variable and overall K6 latent trait score, group membership (i.e., a four category variable representing which cohort an observation was drawn from), and the latent trait score x group membership interaction entered as independent variables in a stepped manner. Model 1 represents a base model with only the latent trait scores predicting the probability of item responses (i.e., no DIF). Model 2 specifies that both the latent trait scores and group membership predict the probability of item responses (i.e., uniform DIF). Finally, Model 3 specifies that both latent trait scores and group membership predict the probability of item responses, but this relationship can vary at different rates of the underlying severity score (i.e., non-uniform DIF). To detect non-trivial levels of DIF, McFadden’s pseudo R^2^ was used to compare the different regression models with a cut-off of R^2^Δ>0.016, a relatively conservative threshold in self-reported health outcomes (Schalet et al., 2020).

**S2. Prevalence of high psychological distress and alcohol use across cohorts and by sex**

**S3. Graphs of the prevalence of high psychological distress across cohorts for those with and without alcohol use by sex**

**S4. Sensitivity Analyses - Heavy Episodic Drinking**

**Table S1: Prevalence of high psychological distress by sex, cohort and presence versus absence of heavy episodic drinking (HED).**

|  | Prevalence of high psychological distress in those *with* HED | Prevalence of high psychological distress in those *without* HED |  | Prevalence ratio within cohorts# | Prevalence ratio across cohorts* |
| --- | --- | --- | --- | --- | --- |
|  | % (95% CI) | % (95% CI) |  | PR (95% CI) | PR (95% CI) |
| *Total sample* |  |  |  |  |  |
| 2007 cohort (ref) | 17.5 (7.9, 27.1) | 2.9 (1.8, 4.0) |  | **6.1 (2.5, 14.7)** |  |
| 2012 cohort | 31.1 (24.6, 37.5) | 7.5 (6.4, 8.6) |  | **4.1 (3.2, 5.3)** | 0.7 (0.3, 1.6) |
| 2014 cohort | 31.0 (20.2, 41.9) | 9.1 (7.9, 10.3) |  | **3.4 (2.3, 4.9)** | 0.6 (0.2, 1.4) |
| 2019 cohort | 42.9 (25.6, 60.1) | 13.1 (11.8, 14.8) |  | **3.2 (2.2, 4.8)** | 0.5 (0.2, 1.3) |
| *Total sample* | *28.5 (23.0, 34.1)* | *9.9 (9.0, 10.7)* |  | *2.9 (2.4, 3.5)* |  |
| *Males* |  |  |  |  |  |
| 2007 cohort (ref) | 12.7 (2.4, 23.0) | 2.9 (1.1, 4.8) |  | **4.3 (1.0, 18.8)** |  |
| 2012 cohort | 24.2 (18.6, 30.0) | 6.7 (5.7, 7.7) |  | **3.6 (2.6, 4.9)** | 0.8 (0.2, 3.3) |
| 2014 cohort | 20.0 (7.0, 33.0) | 5.7 (4.6, 6.7) |  | **3.5 (1.8, 7.0)** | 0.8 (0.2, 3.7) |
| 2019 cohort | 26.3 (9.3, 43.3) | 9.9 (8.1, 11.7) |  | **2.7 (1.4, 5.0)** | 0.6 (0.1, 2,7) |
| *All males* | *19.7 (14.0, 25.4)* | *7.1 (6.2, 8.0)* |  | *2.8 (2.0, 3.8)* |  |
| *Females* |  |  |  |  |  |
| 2007 cohort (ref) | 35.3 (26.7, 43.8) | 2.8 (1.9, 3.7) |  | **12.4 (7.5, 20.6)** |  |
| 2012 cohort | 43.2 (35.6, 50.9) | 8.6 (6.5, 10.6) |  | **5.0 (3.8, 6.8)** | **0.4 (0.2, 0.7)** |
| 2014 cohort | 50.0 (34.9, 65.0) | 11.9 (10.1, 12.7) |  | **4.2 (3.0, 6.0)** | **0.3 (0.2, 0.6)** |
| 2019 cohort | 77.8 (50.5, 105.0) | 17.0 (15.2, 18.8) |  | **4.6 (3.1, 6.7)** | **0.4 (0.2, 0.7)** |
| *All females* | *47.4 (39.9, 54.9)* | *12.6 (11.2, 13.9)* |  | *3.8 (3.2, 4.5)* |  |

# Ratio of prevalence in those with HED compared to those without HED in each cohort

* Ratio of prevalence in the 2012, 2014 and 2019 cohorts compared to the 2007 cohort

Notes: Due to missing data the analysed sample was N=12,823 for high psychological distress and N=13,303 for any HED.

High psychological distress defined as a score of 13+ on the K6.

HED defined as 5+ drinks on a single occasion in the past 6 months (past 3 months for the 2007 cohort).**Table S2. Prevalence of any heavy episodic drinking (HED) by sex, cohort and high versus low psychological distress.**

|  | Prevalence of any HED in those with high psychological distress | Prevalence of any HED in those without high psychological distress |  | Prevalence ratio within cohorts# | Prevalence ratio across cohorts* |
| --- | --- | --- | --- | --- | --- |
|  | % (95% CI) | % (95% CI) |  | PR (95% CI) | PR (95% CI) |
| *Total sample* |  |  |  |  |  |
| 2007 cohort (ref) | 42.4 (21.6, 63.2) | 9.4 (4.8, 14.0) |  | **4.5 (2.3, 9.0)** |  |
| 2012 cohort | 17.6 (12.9, 22.2) | 3.7 (2.8, 4.7) |  | **4.7 (3.5, 6.3)** | 1.0 (0.5, 2.1) |
| 2014 cohort | 4.8 (2.7, 6.8) | 1.1 (0.6, 1.6) |  | **4.3 (2.6, 7.2)** | 1.0 (0.4, 2.2) |
| 2019 cohort | 2.2 (1.0, 3.4) | 0.5 (0.2, 0.7) |  | **4.8 (2.4, 9.5)** | 1.1 (0.4, 2.7) |
| *Total sample* | *6.4 (4.7, 8.2)* | *1.9 (1.3, 2.4)* |  | *3.5 (2.7, 4.5)* |  |
| *Males* |  |  |  |  |  |
| 2007 cohort (ref) | 42.1 (8.6, 75.6) | 13.1 (7.5, 18.7) |  | **3.2 (1.2, 8.7)** |  |
| 2012 cohort | 17.6 (11.6, 23.6) | 4.6 (3.3, 5.8) |  | **3.8 (2.8, 5.4)** | 1.2 (0.5, 3.1) |
| 2014 cohort | 6.9 (2.2, 11.7) | 1.8 (1.1, 2.5) |  | **3.9 (1.8, 8.8)** | 1.2 (0.4, 4.1) |
| 2019 cohort | 2.4 (0.6, 4.2) | 0.7 (0.3, 1.1) |  | **3.2 (1.4, 7.4)** | 1.0 (0.3, 3.4) |
| *All males* | *8.3 (5.1, 11.6)* | *2.8 (1.9, 3.7)* |  | *3.0 (2.1, 4.3)* |  |
| *Females* |  |  |  |  |  |
| 2007 cohort (ref) | 42.9 (25.6, 60.1) | 3.9 (1.7, 6.0) |  | **11.1 (6.8, 18.0)** |  |
| 2012 cohort | 17.6 (10.5, 24.7) | 2.6 (1.6, 3.6) |  | **6.9 (4.8, 9.8)** | 0.6 (0.4, 1.1) |
| 2014 cohort | 3.9 (1.8, 6.0) | 0.6 (0.2, 0.9) |  | **7.2 (3.8, 13.4)** | 0.6 (0.3, 1.4) |
| 2019 cohort | 2.1 (0.7, 2.6) | 0.1 (0.0, 0.3) |  | **16.7 (3.4, 82.1)** | 1.5 (0.3, 7.8) |
| *All females* | *5.3 (3.5, 7.3)* | *0.9 (0.5, 1.3)* |  | *6.0 (4.4, 8.1)* |  |

# Ratio of prevalence in those with HED compared to those without HED in each cohort

* Ratio of prevalence in the 2012, 2014 and 2019 cohorts compared to the 2007 cohort

Notes: Due to missing data the analysed sample was N=12,823 for high psychological distress and N=13,303 for any HED.

High psychological distress defined as a score of 13+ on the K6.

HED defined as 5+ drinks on a single occasion in the past 6 months (past 3 months for the 2007 cohort).

Reference

Schalet, B. D., Janulis, P., Kipke, M. D., Mustanski, B., Shoptaw, S., Moore, R., Baum, M., Kim, S., Siminski, S., Ragsdale, A., & Gorbach, P. M. (2020). Psychometric data linking across HIV and substance use cohorts. *AIDS and Behavior*, 24(11), 3215–3224. PMID: 32430605; PMCID: PMC7873473. https://doi.org/10.1007/s10461-020-02883-5.
